# Learning from silver linings

**DOI:** 10.3389/fnins.2013.00080

**Published:** 2013-05-21

**Authors:** R. McKell Carter, Scott A. Huettel

**Affiliations:** ^1^Center for Cognitive Neuroscience, Duke UniversityDurham, NC, USA; ^2^Brain Imaging and Analysis Center, Duke UniversityDurham, NC, USA; ^3^Department of Psychology and Neuroscience, Duke UniversityDurham, NC, USA

The cardinal goal of decision neuroscience is to identify the neural mechanisms that translate external rewards into an internal sense of value (Rangel et al., 2008; Huettel, 2010; Glimcher, 2011; Rangel and Clithero, 2012). The brain region most commonly associated with “reward” has been the ventral striatum (vStr, neurosynth.org forward inference, 1/2013). Studies of fMRI activation and neuronal activity in the vStr have converged on a standard model in which the vStr computes a reward prediction error [RPE (Schultz, 1997; Pagnoni et al., 2002; O'Doherty et al., 2003; Bayer and Glimcher, 2005)] that then facilitates the learning of new associations (Sutton and Barto, 1998). The RPE mechanism provides flexibility for encoding the relationships between cues and positive rewards across a wide dynamic range, but was historically thought to ignore aversive stimuli (Delgado et al., 2000; Knutson et al., 2001).

A strong split between representations of positively and negatively valued stimuli would be consistent with evidence from classical conditioning (Martin-Soelch et al., 2007) and emotion (Russell, 1980) representation. But, it is inconsistent with a growing body of research that indicates that the vStr can represent the magnitude of value for both positive and negative stimuli (Seymour et al., 2005, 2007; Carter et al., 2009). Research by Brooks and colleagues (Brooks et al., 2010) has pointed toward a way to reconcile this conflicting evidence: negative expectations create a baseline that allows two negative stimuli to be distinguished in the vStr.

Brooks and colleagues presented participants with a series of choices between a constant number of electric shocks and a number based on the outcome of a gamble. The use of primary sensory stimuli in choice tasks is rare but potentially valuable (O'Doherty et al., 2006). Electric shocks in particular provide primary sensory input that is very aversive and can take place in an entirely negative context (i.e., without the use of an endowment). Prior to choice, on each trial, participants were given a standard number of shocks to establish a negative expectation for each trial. The participants' choices were then used to characterize their utility curves. Consistent with prospect theory (Kahneman and Tversky, 1979), the convex shape of these curves indicates that participants, in spite of their preference for fewer shocks, still viewed fewer shocks as a negative outcome. The authors next show that the vStr not only represents aversive choice options, but also does so in a manner that is counter to traditional salience arguments (Blackburn et al., 1992; Salamone, 1994). Less aversive—and therefore less salient—options produce greater activations. This surprising finding has important implications for how associations are learned and subsequent choices are made.

By embedding local context in an RPE framework, Brooks and colleagues can explain two puzzles in the decision neuroscience literature. First, the lack of vStr activation for aversive but unexpectedly positive experiences can now be understood as a problem with firing-rate sensitivity. Negative stimuli that evoke low firing rates can be extraordinarily difficult to distinguish from one another. But, in the local context provided by the negative reference shocks, negative stimuli generate higher overall firing rates and could be easily distinguished.

Second, because these negative stimuli can generate different representations in vStr, the neural machinery responsible for learning positive rewards can be co-opted to choose the more positive of two negative options; widening the applicability of temporal difference models of learning (Sutton and Barto, 1998). The use of local context in temporal difference learning has been described in work on relief from pain (Seymour et al., 2005), and is a potential explanation for striatal representation of monetary losses in one of our own studies where gain and loss contexts were held constant within runs (Carter et al., 2009).

While being able to distinguish negative stimuli in using the vStr expands the applicability of reinforcement models of learning, a number of questions regarding the representation of negative stimuli during choice remain. Work from Hikosaka and colleagues [reviewed in Bromberg-Martin et al. (2010)] has indicated that the vStr may also incorporate signal from dopamine neurons, anatomically distinct in origin, that fire more strongly to both rewards and punishments. Such findings raise the intriguing possibility that a single experiment could reveal anatomically distinct regions within the vStr that evince distinct reward and salience coding—following a clever manipulation of local context. We also note that modern models of decision value have difficulty predicting choices for gambles containing mixed outcomes that include potential gains and losses (Payne, 2005). In order to address these potential shortcomings, choice sets consisting of true mixed gambles may provide important methodological advantages (Venkatraman et al., 2009).

Although much attention has been paid to the representation of aversive stimuli in the vStr, this study by Brooks and colleagues provides an important and novel reminder: subtle differences in experimental protocol can drastically change the neural response to a simple stimulus.
